# Deep Learning in Scaphoid Nonunion Treatment

**DOI:** 10.3390/jcm14061850

**Published:** 2025-03-09

**Authors:** Leyla Tümen, Fabian Medved, Katarzyna Rachunek-Medved, Yeaeun Han, Dominik Saul

**Affiliations:** 1 Department of Trauma and Reconstructive Surgery, Eberhard Karls University Tübingen, BG Trauma Center Tübingen, Siegfried Weller Institute for Trauma Research, 72076 Tübingen, Germany; ltuemen@bgu-tuebingen.de; 2 Department of Trauma and Reconstructive Surgery, Eberhard Karls University Tübingen, BG Trauma Center Tübingen, 72076 Tübingen, Germany; 3 Department of Hand, Plastic, Reconstructive and Burn Surgery, BG Trauma Center Tübingen, Eberhard Karls University Tübingen, 72076 Tübingen, Germany; fmedved@bgu-tuebingen.de (F.M.); krachunek-medved@bgu-tuebingen.de (K.R.-M.); 4 Kogod Center on Aging and Division of Endocrinology, Mayo Clinic, Rochester, MN 55905, USA; han.yeaeun@mayo.edu; 5 Robert Bosch Center for Tumor Diseases, 70469 Stuttgart, Germany; 6 Maybach Clinic, 70469 Stuttgart, Germany

**Keywords:** scaphoid nonunion, fracture healing, nonunion, pseudarthrosis, predictive modeling, deep learning algorithm, TensorFlow, machine learning

## Abstract

**Background/Objectives**: Scaphoid fractures are notorious for a high rate of nonunion, resulting in chronic pain and impaired wrist function. The decision for surgical intervention often involves extensive imaging and prolonged conservative management, leading to delays in definitive treatment. The effectiveness of such treatment remains a subject of ongoing clinical debate, with no universally accepted predictive tool for surgical success. The objective of this study was to train a deep learning algorithm to reliably identify cases of nonunion with a high probability of subsequent union following operative revision. **Methods**: This study utilized a comprehensive database of 346 patients diagnosed with scaphoid nonunions, with preoperative and postoperative X-rays available for analysis. A classical logistic regression for clinical parameters was used, as well as a TensorFlow deep learning algorithm on X-rays. The latter was developed and applied to these imaging datasets to predict the likelihood of surgical success based solely on the preoperative anteroposterior (AP) X-ray view. The model was trained and validated over six epochs to optimize its predictive accuracy. **Results**: The logistic regression yielded an accuracy of 66.3% in predicting the surgical outcome based on patient parameters. The deep learning model demonstrated remarkable predictive accuracy, achieving a success rate of 93.6%, suggesting its potential as a reliable tool for guiding clinical decision-making in scaphoid nonunion management. **Conclusions**: The findings of this study indicate that the preoperative AP X-ray of a scaphoid nonunion provides sufficient information to predict the likelihood of surgical success when analyzed using our deep learning model. This approach has the potential to streamline decision-making and reduce reliance on extensive imaging and prolonged conservative treatment.

## 1. Introduction

A scaphoid fracture is the most commonly fractured carpal bone, accounting for up to 90% of all carpal fractures [1,2], with approximately 1.4–29 fractures per 100,000 person-years in a European population [3]. Anatomically, and due to its limited blood supply and biomechanically crucial role in wrist morphology, the scaphoid is critical for wrist stability, acting as a bridge between the proximal and distal carpal rows. Its anatomical characteristics and reduced blood supply predispose it to complications, particularly in cases of delayed or missed diagnoses. The usually young patient, however, demands a quick return to full function and grip strength [4].

Magnetic resonance imaging (MRI) is regarded as the gold standard in the diagnosis of acute scaphoid fractures, with a reported sensitivity of 94.2% and a specificity of 97.7% [5,6]. Nevertheless, X-rays are frequently utilized in the diagnosis of such fractures due to the accessibility and cost-effectiveness of the procedure [7]. Despite its diagnostic importance and widespread use, X-ray imaging has been demonstrated to exhibit a comparatively low degree of sensitivity (66–81%). Moreover, the diagnosis of scaphoid fractures is complicated by the fact that they can be challenging to detect on initial radiographs, with reported percentages of missed fractures ranging up to 50% [8,9]. This diagnostic uncertainty has led to the development of two main strategies: The use of follow-up imaging (10–14 days later) or advanced modalities such as MRI and CT to complement or replace initial conventional radiography [10]. Another strategy is to enhance the diagnostic value of conventional radiography through the use of artificial intelligence (AI). The advantages of deep learning are the direct use of input data without feature selection, since all available parameters are used, as opposed to logistic regression methods [11]. Machine learning has emerged as a powerful tool, even in modeling complex non-linear, non-stationary processes [12]. These artificial intelligence predictive models have proven superior compared to traditional risk-based models or logistic regression [13,14].

There is a plethora of research investigating the potential of artificial intelligence (AI), particularly convolutional neural networks (CNNs), in detecting fractures in X-ray images [15,16]. Much of this research has evaluated the diagnostic accuracy of these AI algorithms, with findings indicating that they are often comparable to those of clinicians [10]. Research has also indicated that AI algorithms have the capacity to enhance the detection of scaphoid fractures, particularly in cases where the fractures are occult [17,18]. A study by Yoon et al. demonstrated an impressive success rate of 90.9% in the detection of occult fractures of the scaphoid using AI. Consequently, artificial intelligence has emerged as a promising tool in addressing the challenges associated with scaphoid fracture diagnosis [19]. Likewise, CNN models can be trained to extract acoustic signals from joints to classify cartilage degeneration [20] or to predict bone age with higher accuracy compared to regression models [21,22].

Due to the complexity of the clinical diagnosis of scaphoid fractures, the early initiation of appropriate therapy according to established guidelines remains challenging. In case these fractures remain undetected and untreated, a considerable risk of nonunion arises, resulting in significant health-economic burdens and loss of productivity [10]. Untreated scaphoid fractures have been shown to have a nonunion rate of up to 50% [10]. Furthermore, the failure rate of scaphoid nonunion surgery can be as high as 25–40%, which poses a substantial challenge to orthopedic care [23]. Specifically, the scaphoid’s retrograde blood supply renders the proximal pole particularly vulnerable to avascular necrosis, thereby complicating healing and increasing the likelihood of nonunion. Overlooking a clinically unremarkable scaphoid fracture may lead to a nonunion, osteonecrosis, and degenerative arthritis [24].

It is worth noting that the definition of a nonunion remains highly variant, with the majority of clinical definitions characterizing it as the presence of fracture-site tenderness [25]. A commonly cited definition is also the failure of a fracture to unionize within a period of six months [1]. The FDA (United States Food and Drug Administration) considers a nonunion “to be established when a minimum of 9 months have elapsed since injury and the fracture site showed no visibly progressive signs of healing for a minimum of 3 months (no change of fracture callus)” [26]. These nonunions occur in up to 15.5% of all scaphoid fractures, due to insufficient vasculature and frequent delayed diagnoses, outlining this clinical bone’s healing capability as crucial [1,27]. A nonunion can lead to instability, chronic pain, and progressive arthritis, making timely and effective management essential. Nonunions in the scaphoid, a key stabilizer of the wrist, can disrupt carpal mechanics, leading to instability and degenerative changes such as scaphoid nonunion advanced collapse (SNAC-wrist), which can be disastrous for the patient [28]. A SNAC-wrist itself leaves limited surgical options like a partial or complete arthrodesis or proximal row carpectomy, with or without scaphoid excision [28].

A scaphoid nonunion alone frequently requires bone grafting (vascularized or nonvascularized) and precise fixation to restore anatomy and function. Choosing the most appropriate intervention is challenging, especially in cases of avascular necrosis or severe deformity. Subsequently, different classification systems for scaphoid nonunions have been developed to deduce the next therapeutic step, such as the classification by Filan and Herbert from 1996 [29], the classification by Slade and Geissler [30], or Slade and Dodds [31].

Despite surgical and radiological advancements, achieving consistent union and functional recovery remains difficult, particularly in complex cases. Recent advancements in artificial intelligence (AI) might counter these intricate challenges of diagnosing and treating scaphoid nonunions. AI has the potential to revolutionize care by analyzing large datasets to identify patterns and predict outcomes, as it can assist in reliably selecting operative approaches by evaluating factors such as fracture location, vascularity, patient-specific anatomy, and imaging features. The latter solely relies on specific radiological features without any additional patient data. This data-driven insight can guide surgeons in making evidence-based decisions, improving outcomes, and minimizing complications in this challenging clinical scenario. Consequently, the development of strategies for the early and accurate diagnosis of scaphoid fractures is imperative. To address these challenges, artificial intelligence has been developed to predict the occurrence of nonunion based on X-ray images. This innovation has the potential to represent a significant advance in the prevention and treatment of scaphoid fractures and serve as a decision-making aid in the event of impending pseudarthrosis. By employing artificial intelligence (AI) technology to predict pseudarthrosis at an early stage, there are a number of benefits for treatment, including the ability to individualize treatment strategies and reduce unnecessary immobilization or invasive procedures. Key advantages of AI include consistency, reproducibility, and sensitivity. It reduces interobserver variability, common among human experts [32], and its standardized interpretations can be particularly valuable in cases where specialized radiologists are not available. Furthermore, AI can significantly enhance the sensitivity of relevant radiological features that can be overlooked by human eyes [33]. In fact, multiple studies have proposed AI models for fracture detection, presenting high diagnostic accuracies comparable to human clinicians [34,35,36] or surpassing them in some cases [37].

Machine learning (ML) is a branch of AI that has been widely used in the field of disease diagnosis and prognosis [38,39,40]. Traditional ML classifiers, such as logistic regressors or support vector machines, require manual feature extraction, making them dependent on predefined hypotheses [10]. In contrast, deep learning can autonomously learn hierarchical features, rendering them highly effective at capturing nonlinear correlations [41].

Hence, the aim of this study was to use only plain X-ray images of scaphoid nonunions in a large database of nonunion patients and train a deep learning algorithm as well as a logistic regression to reliably identify the class of nonunion cases with a high chance of later union after operative revision.

## 2. Materials and Methods

**Figure 1:**
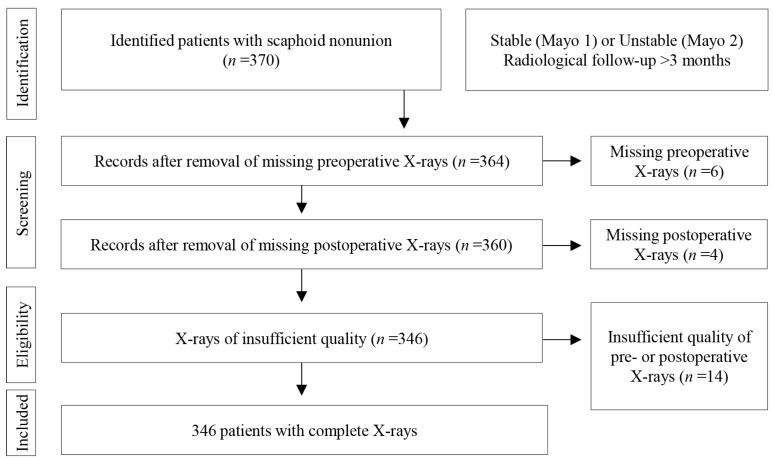
Parameters of inclusion for study patients. After initially identifying 370 eligible patients, 6 patients were excluded due to missing preoperative X-rays and 4 due to missing postoperative X-rays. Upon further examination of each individual X-ray, 14 were excluded due to insufficient quality.

Initially, 370 patients were identified with scaphoid nonunion from January 2007 to December 2020. After applying exclusion criteria such as missing complete pre- and postoperative X-rays or insufficient quality, *n* = 346 patients remained (Figure 1).

After established nonunion, osteosynthesis was performed after a bone graft was implemented. The bone grafts used were nonvascularized from the iliac crest, vascularized pedicled bone grafts from the distal radius, or vascularized free grafts from the medial femoral condyle.

We first used logistic regression with scikit-learn (1.6.1) in Python 3.8 (Python Software Foundation, Wilmington, DE, USA) with the parameters “intraoperative avascular necrosis”, “smoking status”, “age”, “sex”, and “side” to determine the treatment outcome.

**Figure 2:**
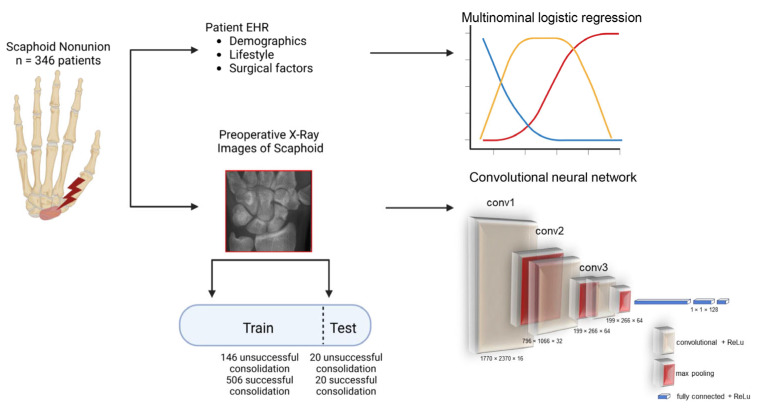
Overview of the study. After selecting *n* = 346 scaphoid nonunion patients, the patient demographics, lifestyle, and surgical factors were used for a multinominal logistic regression or split into a training vs. test dataset in order to create a convolutional neural network.

Subsequently, for the deep learning algorithm, we analyzed 83 anteroposterior X-rays of the preoperative wrist that did not heal properly, as well as 263 anteroposterior X-rays that healed properly. The details of our analyzed cohort have been described by Rachunek-Medved et al. elsewhere [42]. An overview of the overall study design is shown in Figure 2.

The whole dataset was split into a training dataset (unsuccessful: 146 images, successful: 506 images, totaling 652 images) and a test dataset (unsuccessful: 20 images, successful: 20 images, totaling for 40 images), based on the recommendations of Joseph [43].

To build the deep learning model, TensorFlow 2.3.0 (Google LLC, Mountain View, CA, USA) with numpy 1.20 (NumPy Developers, Berkeley, CA, USA) for Python 3.8 was used. For visualization purposes, matplotlib 3.7.5 (Python Software Foundation, Wilmington, DE, USA) was utilized. Following the recommendations of Brown et al. [44], and Busfield on image classification [45], a convolutional neural network was trained on 1770 × 2370 pixel grayscale X-ray images. Images were classified as ‘successful’ or ‘unsuccessful’ based on whether the surgical outcome was ‘healed’ or ‘not healed’ after at least three months, as described in detail by Rachunek-Medved et al. [42]. In short, a training dataset and validation dataset were created and manually rescaled by dividing by 255 (as grayscale channel values lie between 0 and 255). Subsequently, convolutional layers, mixed with pooling layers, were created in gradually downsized scales, and the model itself unwound to 1D, before deploying a series of dense layers, ensuring the number of nodes matched the number of possible classifications (*n* = 2) (Figure 2). The preprocessing layers, core layers, and dense layers were then sequentially used to generate a model with Keras APIs via TensorFlow. The training algorithm was stopped as soon as validation loss was not improving any more, with a maximum of 20 epochs.

Approval from the Institutional Review Board (number 560/2020B0) was obtained from Eberhard-Karl University in Tübingen. Patients with scaphoid reconstruction between January 2007 and December 2020, with a follow-up of at least three months, were included. Screening was conducted using the CGM-MEDICO HIS software (v28) and involved ICD-M84.14 (pseudarthrosis/nonunion) of the hand.

## 3. Results

**Figure 3:**
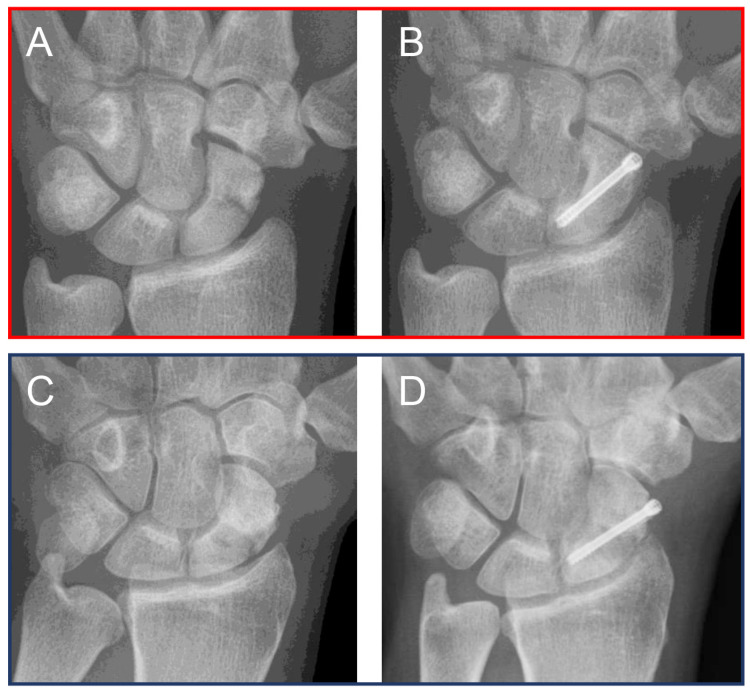
Examples of successfully (red frame) and unsuccessfully healed (blue frame) scaphoid nonunions. (**A**) A scaphoid nonunion with a nonunion in the scaphoid waist, D2 by Filan and HBS [29], grade 3 by Slade and Geissler [30]. (**B**) Completely consolidated scaphoid nonunion after bone graft plus HBS. (**C**) A scaphoid nonunion with a waist fracture, D3 by Filan and HBS, grade 4 by Slade and Geissler. (**D**) Missed bone union after attempted reconstruction with a bone graft plus HBS.

We analyzed 346 patients with scaphoid nonunions, divided into 83 cases of failed scaphoid reconstructions and 263 successful scaphoid reconstructions based on union outcomes. Scaphoid nonunions presented with clinically evident pain at the wrist, mostly in the foveola radialis/anatomical snuffbox (Figure 3A), and were treated surgically predominantly with bone grafts and a headless bone screw (HBS), before they healed completely (Figure 3B). In other cases, a similar clinical presentation (Figure 3C) yielded a similar clinical treatment with an HBS but did not result in a healed state (Figure 3D).

These two groups (union vs. non-union) significantly differed in smoking status and intraoperative avascular necrosis, as unsuccessful operations had a higher proportion of smokers and intraoperative necrosis. In addition, the used grafts did not differ significantly (Table 1).

The logistic regression led to an accuracy of 66.30% (precision = 81.22%, recall: 72.52%, F1: 0.7580), with intraoperative avascular necrosis as the largest factor, and sex and side as redundant factors (Table 2, performance: Supplementary Figure S1A,B). 

**Figure 4:**
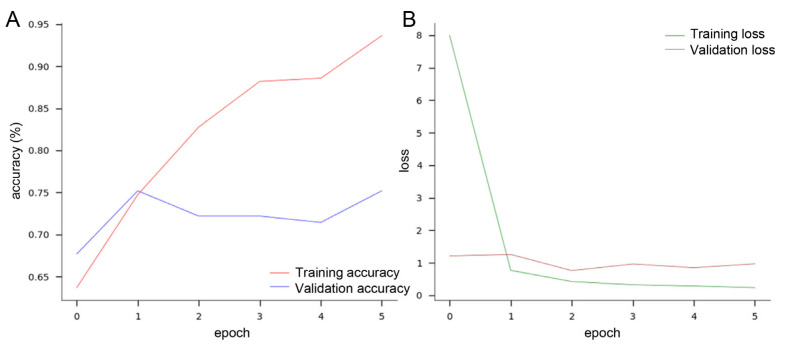
Results from TensorFlow deep learning model. (**A**) After five epochs, a training accuracy of 93.63% was achieved (red line) with a validation accuracy above 70% (blue line). (**B**) The training loss was below 1 after one epoch (green line), slightly lower at the fifth iteration with a validation loss constantly around 1 (brown line).

After randomly assigning anteroposterior X-ray images to the training or test dataset, we used a TensorFlow algorithm to train multiple layers of the deep learning algorithm on these 652 and 40 images, respectively. After five epochs of iterative analyses, the training accuracy reached 93.63%, with a training loss below 1 (Figure 4A,B, performance: Supplementary Figure S1C,D).

We performed a post-hoc power analysis with a sample size of *n* = 346, effect size of ρ = 0.2 and α-error = 0.05, resulting in an achieved power of 0.98 (G*Power 3.1.9.7, University Kiel, Kiel, Germany).

## 4. Discussion

The scaphoid, as the most commonly fractured carpal bone, continues to present significant challenges in clinical practice. Its anatomical and vascular peculiarities predispose it to complications, particularly when early diagnosis is missed. Alarmingly, up to 25% of patients with scaphoid fractures seek medical attention only after six months, often when the fracture has progressed to a nonunion state [46]. Factors such as diabetes, comminution, and the development of a humpback deformity further exacerbate the risk of nonunion, often prolonging the healing process and complicating treatment [47,48].

This situation is particularly concerning in young, active patients who depend on the swift restoration of wrist function and grip strength. Contemporary management of scaphoid nonunions has increasingly shifted towards arthroscopic techniques combined with bone grafting [49]. However, the presence of osteonecrosis frequently complicates the healing trajectory, making treatment outcomes less predictable [50]. Notably, postoperative MRI, though commonly employed, has shown limited validity in assessing avascular necrosis in these cases, underscoring the need for more reliable diagnostic and predictive tools [7]. A logistic regression is usually applied as a multivariable method to model dichotomous outcomes [51], and has performed with an accuracy of 66.3% in this study. However, the performance of a logistic regression compared to a machine learning approach, even if only applied to sole X-rays, may be inferior [52].

While convolutional neural networks (CNNs) have demonstrated exceptional accuracy in detecting scaphoid fractures using conventional X-rays [53], their application in predicting treatment success remains relatively unexplored. This study addresses this gap by leveraging a robust dataset of 346 patients with scaphoid nonunions, all of whom had both preoperative and postoperative X-rays. Using a TensorFlow-based deep learning algorithm, this study successfully predicted surgical outcomes with a remarkable accuracy of 93.63%, relying solely on preoperative anteroposterior (AP) X-rays. This innovative approach highlights the potential of AI to revolutionize decision-making in this challenging clinical domain.

The findings align with prior research emphasizing the multifactorial nature of scaphoid nonunion outcomes. A multicenter study involving 138 patients with established scaphoid nonunions revealed that approximately 20% of the patients experienced persistent nonunion after surgical interventions. Factors such as heavy labor, nonunions persisting for over five years, extended immobilization periods, and delays in treatment following initial trauma significantly reduced healing probabilities [54]. These insights underscore the importance of timely and tailored interventions.

In addition to the scaphoid, machine learning has demonstrated promise in predicting nonunion in other contexts. For instance, a study using MRI images from 505 patients with vertebral fractures showed that machine learning models outperformed traditional methods like logistic regression and decision trees in terms of reliability and accuracy to predict nonunion [55].

These advancements suggest a broader applicability of AI in orthopedic care, offering new avenues for personalized and precise treatment strategies—outperforming classical logistic regressions. The integration of AI into clinical practice heralds a transformative era in the management of scaphoid nonunions. By harnessing the predictive power of deep learning, clinicians can make more informed decisions, reducing reliance on invasive diagnostics and streamlining the treatment process. Future research should focus on expanding these models to include diverse imaging modalities and patient-specific variables, further enhancing their predictive capabilities. However, there are various challenges when it comes to implementing this technique in the clinical workflow: While hardware limitations and the use of different formats might be technically resolvable, legal questions arise: Who is responsible for a change of primary care? As the used model works as a “black box”, some results may be correct in their prediction potential, but it is not evident on which factors/features these assumptions are based [56,57]. Nonetheless, the TensorFlow-based model developed in this study not only exemplifies the potential of AI in improving surgical planning but also marks a significant step toward the realization of personalized medicine in orthopedics.

Limitations of this study are the sole use of X-rays, with no addition of patient-specific characteristics (smoking, non-smoking, age, comorbidities, quality of bone graft, etc.), which was chosen to provide a non-biased, pure radiological predictor. Additionally, more parameters, like initial fracture displacement, time since injury, and fracture type, could have enhanced the model’s predictive accuracy, but have not been recorded consistently. The analyzed dataset is relatively small, but a scaphoid nonunion is usually referred to special centers, as in these cases, and higher numbers are difficult to obtain. A comparison to other machine learning approaches like Grad-CAM or support vector machines (SVMs) could highlight the classification features, and a correction for radiographic artifacts might have improved the accuracy.

## 5. Conclusions

In the present study, a novel deep learning model was utilized to predict surgical success in the treatment of scaphoid nonunions. The model has been shown to be a reliable tool that identifies scaphoid nonunions with an accuracy of 93.63%, solely based on the preoperative radiographs. The implementation of this AI-based analysis method in clinical practice has the potential to prove beneficial through two main mechanisms. Firstly, it offers the promise of enhancing the efficiency of decision-making processes. Secondly, it could potentially reduce reliance on extensive diagnostic imaging and prolonged conservative treatment, leading to more efficient management of patients.

Despite the encouraging findings, there are certain limitations that must be acknowledged. Our model is solely based on AP radiographs and does not take into account other potentially relevant imaging modalities, such as computed tomography, nor patient-specific characteristics, including smoking status and age. Addressing these limitations through the incorporation of additional imaging modalities and patient-specific characteristics in subsequent research may enhance the precision of the prediction model.

## Figures and Tables

**Table 1:** Characteristics of the unsuccessful and successful healing groups.

	Successful Operation (*n* = 263)	Unsuccessful Operation (*n* = 83)	*p*-Value
Age (yrs) [mean, ±SD]	34.6 ± 12.0	32.9 ± 10.3	0.25 ^1^
Sex (female) [n, %]	26, 9.9%	5, 6.0%	0.38 ^2^
Side (right) [n, %]	127, 48.3%	40, 48.2%	>0.99 ^2^
Smoking status (yes) [n, %]	61, 23.2%	38, 45.8%	0.0001 (***) ^2^
AVN intraop. (yes) [n, %]	27, 11.6%	21, 36.8%	<0.0001 (****) ^2^
Grafts used [n, %)			
Non-vascularized	191, 72.6%	57, 68.7%	0.49 ^2^
Vascularized, pedicled	59, 22.4%	23, 27.7%	0.37 ^2^
Vascularized, free	11, 4.2%	3, 3.6%	>0.99 ^2^
None	2, 0.8%	0, 0%	>0.99 ^2^

^1^ Unpaired *t*-test, ^2^ Fisher’s exact test. A p value less than 0.05 is considered as statistically significant (*** *p* < 0.001, **** *p* < 0.0001).

**Table 2:** Parameters of the logistic regression model.

Features	Coefficients
AVN intraop. (yes)	−0.50
Smoking	−0.15
Age	0.01
Sex	0.00
Side	0.00

## Data Availability

The raw code can be accessed upon reasonable request.

## Supplementary Materials

The following supporting information can be downloaded at: https://www.mdpi.com/article/10.3390/jcm14061850/s1. Figure S1: Performance of logistic regression and deep learning. (A) Confusion matrix of logistic regression, (B) ROC Curve of logistic regression, (C) Confusion matrix of deep learning model, (D) ROC Curve of deep learning model.
