# Supplementary figures and images for: Deep Learning in Scaphoid Nonunion Treatment
Source: J Clin Med. 2025 Mar 9;14(6):1850. doi: 10.3390/jcm14061850 (PMC11942999; doi:10.3390/jcm14061850)

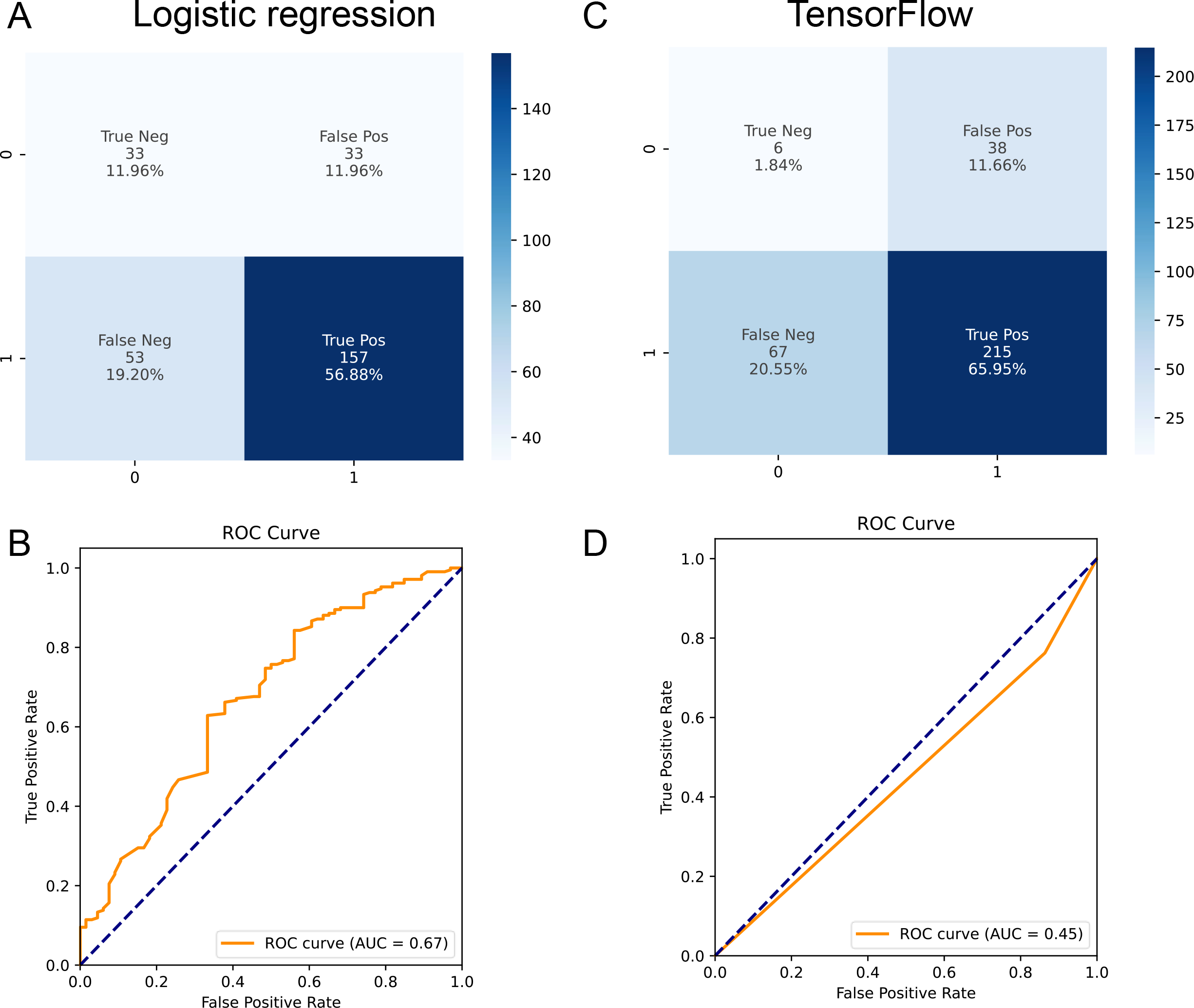

Supplement: Supplementary file 1 [file jcm-14-01850-s001.zip › jcm-3497299-supplementary.tif]
